# Challenges to Improving Health Care Delivery Through ERISA Fiduciary Litigation

**DOI:** 10.1017/jme.2026.10231

**Published:** 2026

**Authors:** Norman Stein, Elizabeth Hopkins

**Affiliations:** 1 Drexel University Thomas R Kline School of Law, United States; 2 Hopkins ERISA Law, Los Angeles, California, United States

**Keywords:** ERISA, fiduciary, employer-provided health care, Pharmacy Benefit Managers, prohbitied transactions

## Abstract

Commentary on “ERISA and the Failure of Emplloyers to Perform Their Fiduciary Duties: Evidence from a Survey of Health Plan Administrators.”

## I. Introduction

Almost a quarter century ago, the Department of Labor’s Advisory Council on Employee Welfare and Pension Plans issued a report on “Challenges to the Employment-Based Healthcare System,”^1^ an approach to health care delivery that scholars have characterized as unique among advanced nations.^2^ The report included this fundamental conclusion: “… if we were starting with a blank slate, we would learn from the problems facing the current system and design a new paradigm that is very different from today’s model. For example, in designing a system from scratch, the majority of the committee would probably favor basic medical care for all under a federal system, with additional or private care available on top of that from non-governmental funding.”^3^

The report, despite its pessimistic take on employer-provided health care plans generally, did identify some positive features of the system over individually obtained insurance policies, particularly for employees of larger employers, including reducing per-capita administrative expenses through economies of scale, the natural risk pooling provided by a large workforce, and the enhanced bargaining position of an employer over an individual in negotiating with service providers. Under ERISA, the employer’s selection process for the third parties who will provide services for the plan, the negotiation of contracts with such parties, and the monitoring of their performance are typically subject to the statute’s fiduciary standards.

The paper that is the subject of this commentary, “ERISA and the Failure of Employers to Perform Their Fiduciary Duties: Evidence from a Survey of Health Plan Administrators,” is focused on how well employers perform these fiduciary functions. Based on data from an innovative survey of how employers perform these functions, the authors of the paper conclude that most employers fail to meet their basic statutory obligations as fiduciaries under ERISA when hiring third-party administrators (TPAs), typically insurance companies, and other service providers to administer benefits under their health care plans. Although two-thirds of the surveyed employers requested bids or offers from multiple plan administrators and attempted to develop appropriate benchmarks to compare service providers, and almost half negotiated with third-party providers over price, more granular survey questions suggested that even these employers generally failed to gather sufficient relevant information to allow them to evaluate adequately the quality and comparative cost of services they were purchasing. For most employers, ongoing monitoring failed to include surveying employee opinion on either quality or cost.

This is valuable information. On this basis, the authors conclude, and we agree, that many employers fail to perform their selection and oversight functions in accordance with ERISA fiduciary standards.^4^ And this negates the primary theoretical virtue of employer-provided health care: the employer’s ability to use its resources and superior bargaining power to act as an effective agent for its employees.^5^

The authors, however, do not call, at least expressly, for major structural reform of employer-provided health care, but rather focus on employers improving their compliance with ERISA fiduciary standards. There are two related legs to their argument: first, that concern about potential ERISA fiduciary liability for shortcomings in their fiduciary performance will motivate employers to improve performance of those functions; and second, that the improved performance will meaningfully address the problems of employer-provided health care. While we fully agree with the authors that many employers are failing to satisfy their ERISA fiduciary duties, we are at least somewhat skeptical that employers will be nudged into better behavior because of fear of liability. More important, we are skeptical that improved fiduciary performance by employers will adequately address the “inefficiencies across the broader national market” for health care. Here we suspect the authors would agree with us that improving employer fiduciary performance alone is an incomplete response to the problems of health care delivery in the United States. To the contrary, we think better oversight by employers is unlikely to appreciably improve health care delivery or have more than a marginal impact on cost containment for a number of interrelated reasons. We briefly discuss each of these topics.

In an afterword to the paper, we provide some thoughts on potential litigation against TPAs, insurers, pharmacy benefit managers (PBMs), and other third parties who are in every real sense responsible for the operation of a plan. While it is unclear that such suits would succeed, success here has some potential to improve markedly the efficiency, quality, and cost of employer-provided health care plans.

## II. Fear of Liability

As the authors note, participant-initiated litigation and Department of Labor (DOL) enforcement actions against employers for failure to satisfy their fiduciary obligations in the health care space — i.e., with respect to selecting and negotiating with third parties to administer and manage their health care plans and then periodically monitoring their performance — has been less frequent than in the retirement space. If such litigation were more frequent and successful, presumably recalcitrant employers would become more proactive, if only to avoid potential litigation and financial liability.

But there are reasons that employers are either unaware of, or alternatively do not fear the consequences of ERISA enforcement for the fiduciary failures identified in the paper. First, as the authors note, the Department of Labor has not historically focused enforcement or other regulatory attention on the employers’ fiduciary functions in health care plans. Even accepting the authors’ cautious optimism that the DOL may become more active in this area, given the recent, substantial reduction in DOL staff and the time-consuming and uncertain nature of health care litigation, any such litigation might not have a significant impact on employer behavior, at least in the short term.

Second, fiduciary litigation against employers has been relatively sparse in the ERISA health care area, not because attorneys are unaware of ERISA, but because they are all too aware of the substantial obstacles for plaintiffs to successfully litigate in this area: standing,^6^ class certification,^7^ pleading,^8^ settlor/fiduciary distinction,^9^ and damage and remedial issues.^10^ Many recent cases have been dismissed on one or more of these bases, and even cases that proceed successfully to trial are sometimes partially or completely reversed on appeal.^11^ Thus, the obstacles to bringing a successful claim are great, and the plaintiff’s litigation costs can be substantial. It is not very surprising, then, that there has been limited private civil litigation challenging the employer’s fiduciary performance in selecting and overseeing TPAs and other service providers in the health care area.^12^ All things considered, there is reason to be skeptical that employers will suddenly become better agents for their employees out of fear of DOL enforcement or private litigation. This is especially true for small employers, who would be unlikely targets for plaintiff attorneys, at least initially.

We can also offer some speculation about why ERISA’s fiduciary standards have not resulted in better health plan offerings to date. We think it unlikely that the main driver of poor health care plan performance relative to price is that employers do not understand their duties or appreciate their potential liability. A greater problem is likely that the pricing and payments systems among the insurers and health care middlemen, such as PBMs, are incredibly (and perhaps intentionally) complex and opaque, and information about pricing and fees is difficult, if not impossible, to come by, despite some recent legislation noted by the authors.^13^ The authors note that recent legislative changes may make information about pricing and comparative pricing easier to come by; however, the legislative changes they identify, while significant, do not provide complete cost transparency.

The employer fiduciary also faces a problem of market consolidation: in many markets there are only a handful of insurers who provide health insurance or administrative services for health care plans.^14^ For similar reasons, the product offerings among the third parties who administer health care plans are not sufficiently varied, depriving even a vigilant fiduciary of wide-ranging choice in the type of products on the shelf.^15^ This is not true with respect to retirement plans, for which there is a more varied marketplace and a wider range of product types.

Another potential problem for the employer is the diversity of interests among the participants. A plan with limited choice of health service providers, a limited pharmaceutical formulary, and high deductibles will be attractive to some plan participants (those who are currently healthy, relatively cost conscious, and favor lower employee premiums), but not to others. Similarly, the choice between higher premium contribution or higher deductibles will have disparate appeal to different participants.

Moreover, the employer itself will often have an interest in reducing overall costs. And while a particular employer’s preference for cost reduction over higher quality care, as we suggested in the prior paragraph, may align with the interests of some participants, it likely would not be in the interests of many others. In any event, it is unclear the extent to which such matters are ultimately a matter of plan design, and thus settlor functions, rather than fiduciary decisions subject to ERISA’s standard of care and loyalty.

## III. Are ERISA Fiduciary Standards the Path to Improving Our Health Care System?

Assume that our views in Part II are wrong, that a wave of health care litigation is on the horizon and that the eventual upshot will be that employer fiduciaries will revise their procedures for selecting and monitoring third-party health care plan providers. Would that lead, as the authors posit, to correcting “inefficiencies across the broader national market?” We have two concerns: first, that consolidation in the health care industry will limit the likely impact of improved fiduciary processes, since the current marketplace limits choice. As we already noted, alternatives to the costly and often poorly functioning products currently available in the health plan marketplace are in short supply. And the settlor function of designing an affordable plan, echoed by the cost-conscious preferences of many plan participants, may further constrain the amount of possible overall improvement.

Our second concern is that of unintended consequences. If employers have significant concerns about potential liability in response to participant civil actions or vigorous DOL enforcement activity, they have at least two choices: improve their fiduciary processes or get out of the business of providing health care plans. The ACA does not require employers of fewer than 50 employees to provide a health care plan, and as the authors note, employers have the option of sponsoring an Individual Coverage Health Reimbursement Account. Since the enactment of ERISA, we have seen a stunning shift in employer-sponsored retirement plans from defined benefit pension plans to 401(k) plans. Perhaps the combination of ERISA and the ACA will result in a similar shift from employer health care plans to individuals selecting their own plans from the ACA marketplace. This may not be a path to a more effective health care delivery system.

We move from this gloomy outlook to note other possible pathways to improving our health care system. As we have noted, the few nationally available TPAs are mostly insurance companies, which have begun to vertically integrate by merging with pharmacy benefit managers, pharmacies, and hospitals, as well as other medical practices. Perhaps we should turn to antitrust-type solutions, breaking up the concentration of health plan suppliers in the hopes that this will spur innovation and better plan designs. Another option would be to set up a public alternative to private sector health care plans. Or perhaps, as the DOL Advisory Council suggested in 2002, we might consider establishing a federal catastrophic insurance program covering all Americans, which would address a number of problems with our current system and might make solving other problems easier to manage.

Here we return to the paper, which does not claim — and we do not believe it intended to claim — that more robust compliance by employers with ERISA’s fiduciary duties is the most promising theoretical path to reforming health care delivery in the United States. But the types of reforms that we suggested in the last paragraph, and the more extreme alternative of moving to the universal health care model used by most industrialized nations, face forbidding political obstacles. Even assuming that those obstacles could be overcome, each of these potential reforms would create transition problems and almost certainly their own unintended consequences. If so, the authors may well be right in their prescription, imperfect as it may be. If it is the only available road or the most available pathway to at least some reform, we may need to take it. Removing obstacles to ERISA litigation through legislative or regulatory reform would help. But there are no easy fixes given the complex health care system that exists in our country today, and it is worth trying to map more than one road to better outcomes as we try to improve our nation’s delivery of health care.

## IV. Afterword: Some Additional Thoughts

For us, a problematic aspect of the paper is that its primary focus is on the employer’s duties as an ERISA fiduciary. As we suggest in this short commentary, the employer occupies a difficult space: choosing and contracting with service providers but typically lacking the expertise and experience to do so effectively, especially in a maddeningly complex and opaque health care marketplace. The entities with the capacity to create more inclusive, better-value health care for participants in employer-provided health care plans are the third parties that administer and manage health care plans: typically insurance companies, as we have mentioned, as well as pharmacy benefit managers and other middlemen, which are sometimes but not always associated with the insurance companies. Yet, as with litigation against fiduciary employers, there are numerous, similar barriers to fiduciary lawsuits against TPAs and other plan service providers.

Moreover, unlike employers and TPAs, PBMs and other service providers to health care plans are sometimes not considered ERISA fiduciaries at all. Regulatory changes making clear that entities such as PBMs are fiduciaries when they engage in certain plan-related activities would make them susceptible to civil enforcement actions that might change their practices in helpful ways.

Furthermore, mandating additional, detailed and easy-to-understand disclosures about fees and health care payment and processing practices, and placing the onus for this disclosure on the TPAs and service providers that have the information, is an essential piece of the solution.^16^

In addition, legislative changes making clear that the Department of Labor may enforce fiduciary requirements against insurers that act as TPAs for health care plans would be helpful.^17^

Finally, it is worth noting that ERISA’s prohibited transaction provisions provide a powerful mechanism for ensuring that plans are not overcharged by plan service providers and that the assets of health plans are used, as intended, for providing benefits to plan participants and beneficiaries.^18^ Ensuring that these provisions remain available to private litigants is an essential piece of the equation. In this regard, we caution that enacting recent legislative proposals designed to make it harder for private litigants to plead a prohibited transaction violation^19^ would be counterproductive to attempts to utilize ERISA litigation to reduce health care costs and improve private health care plans.
